# IgG4-Related Laryngeal Disease as a Possible Cause of Idiopathic Subglottic Stenosis: A Case Report

**DOI:** 10.22038/ijorl.2020.47106.2547

**Published:** 2021-03

**Authors:** Ádám Bach, Andrea Ambrus, Béla Iványi, Zoltán Tóbiás, Gholam Hossein Alim Marvasti, László Rovó

**Affiliations:** 1 *Department of Otorhinolaryngology & Head- Neck Surgery, University of Szeged; Tisza Lajos krt. 111., 6725, Szeged, Hungary.*; 2 *Department of Pathology, University of Szeged, Állomás u. 2, 6725, Szeged, Hungary.*

**Keywords:** Fibro-inflammatory disorder, Idiopathic subglottic stenosis, IgG4, Laryngotracheoplasty

## Abstract

**Introduction:**

Immunoglobulin G4-related disease (IgG4-RD) is a systemic fibro-inflammatory disorder. Laryngotracheal manifestation is very rare; therefore, it is usually associated with complex diagnostic and therapeutic problems.

**Case Report:**

Herein, we report the case of a 35-year-old woman with idiopathic subglottic stenosis (ISGS) treated with one-step laryngotracheal reconstruction surgery. Postoperatively, the lesion was found to be a part of the IgG4-RD spectrum. Objective and subjective phoniatric tests, spirometry, and Quality of Life Questionnaire were used for the evaluation of postoperative functional results. Slide laryngotracheoplasty as a one-step surgery without stenting and tracheostomy ensured a sufficiently wide subglottic space with no adverse effect on voice quality. During a follow-up period of 22 months, endoscopy and computed tomography scan revealed no significant restenosis. The patient was able to return to premorbid activities of daily living without any further medical treatment.

**Conclusion:**

The laryngeal involvement of IgG4-RD is uncommon; however, it is a manifestation that should be included in the differential diagnosis of subglottic stenoses (SGS). Furthermore, subglottic IgG4-RD might be a potential etiological factor of ISGS and acquired airway stenosis after short-term intubation. Slide laryngotracheoplasty might be a favorable solution without stenting and tracheostomy even in special cases of SGS.

## Introduction

Immunoglobulin G4-related disease (IgG4-RD) is a rare immune-mediated fibro-inflammatory disorder of unknown origin with heterogeneous pathologic features and variable disease manifestations. IgG4-RD is a systematic condition characterized by abundant IgG4 positive plasma cell infiltration and tumor-like lesions of the involved organs and consequently is often misdiagnosed as either malignancy or granulomatous conditions (1-4). Single organ manifestations have already been described more than 125 years ago; nonetheless, a consensus on description and nomenclature was achieved only in the last decade (5). Autoimmune pancreatitis represents the prototype of IgG4-RD; however, IgG4-RD may affect virtually any organ (2). The interest in IgG4-RD has considerably grown in the past few years and extended to specific disorders across most medical subspecialties. A broader understanding of IgG4-RD has led to the recognition that many medical conditions long viewed as unique diseases are part of the IgG4-RD spectrum. The expanding list of organs involved in IgG4-RD includes the pancreas (autoimmune pancreatitis), biliary ducts (sclerosing cholangitis), kidneys (tubuleinterstitial nephritis), meninges, aorta, lungs, prostate, breast, pericardium, pituitary gland, lymph nodes, and skin (1,4,6). The head and neck region, especially the orbits (chronic sclerosing dacryoadenitis and inflammatory orbital pseudotumor), paranasal sinuses, major salivary glands (Küttner’s tumor), and thyroid gland (Riedel’s thyroiditis) are among the most frequent areas of involvement (3). Nevertheless, the laryngotracheal manifestation is very rare; therefore, it is usually associated with complex diagnostic problems. Herein, we report a patient with idiopathic subglottic stenosis (ISGS) treated with one-step laryngotracheal reconstruction surgery. Postoperatively, the lesion was found to be a part of the IgG4-RD spectrum. This case emphasizes the necessity to contemplate IgG4-RD in the differential diagnosis of ISGS and simultaneously demonstrates a favorable surgical solution for the problem.

## Case Report

A 35-year-old Caucasian woman presented with an 8-year history of progressive dyspnea on exertion. She had no history of prolonged intubation, airway trauma, or gastroesophageal reflux disease (GERD) and was a lifetime non-smoker. Apart from insulin-dependent diabetes mellitus, she had no other chronic disease. 

**Fig 1:**
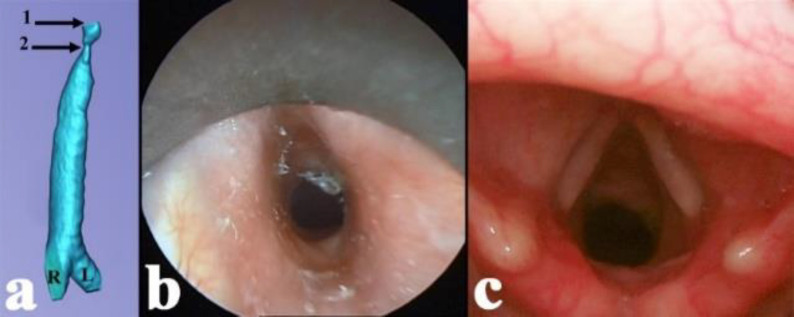
**a)** three-dimensional computed tomography reconstruction of the air shadow (preoperative). 1: glottic level; 2: subglottic stenosis; R: right main bronchus; L: left main bronchus. **b)** direct preoperative endoscopic picture of the stenotic airway; the subglottic airway covered by a metaplastic exfoliative squamous epithelium.**c)** direct endoscopic picture of the larynx during inspiration (22^nd^ postoperative month)

**Fig 2:**
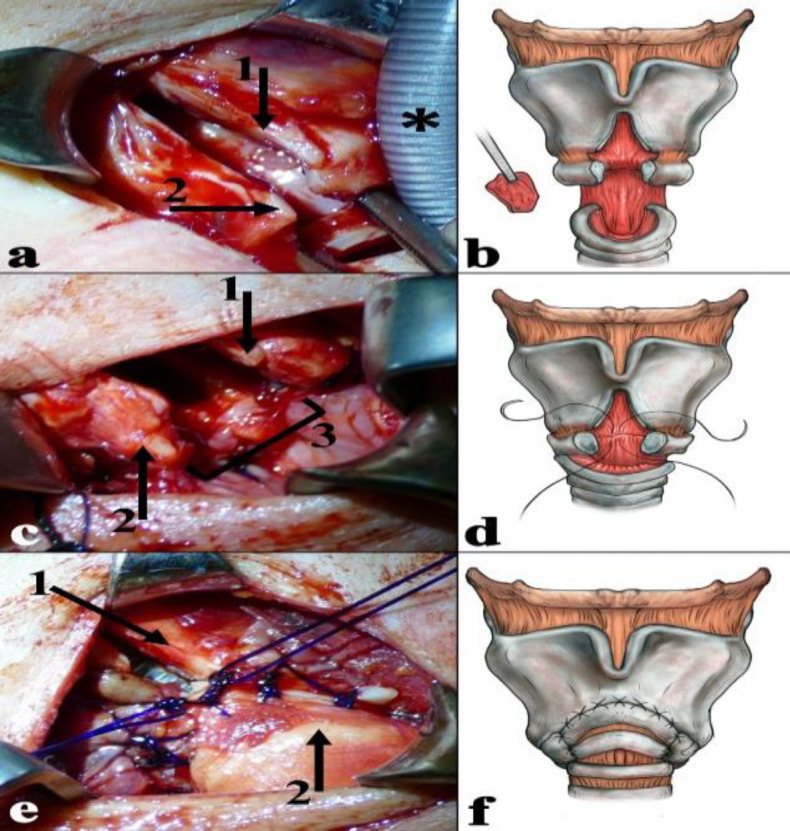
a) partial anterior laryngofissure; 1: incised thyroid cartilage; 2: cricoid cartilage; *: endotracheal tube. **b)** partial anterior laryngofissure with the preservation of the anterior commissure of the vocal folds; partial resection of membranous part of the trachea.** c)** reconstruction of the posterior wall; 1: thyroid cartilage; 2: cricoid cartilage; 3: anastomosis of the posterior wall. **d)** formation of an anastomosis between the trachea, cricoid, and thyroid; firstly, reconstruction of the posterior wall. **e)** reconstruction of the anterior wall; 1: thyroid cartilage; 2: second tracheal ring. **f)** reconstructed anterior and lateral wall

The breathing problem started after a septorhinoplasty under general anesthesia, performed due to a spontaneous deformation of the cartilaginous nasal septum. To identify a supposed underlying autoimmune disease, blood tests and histopathologic examinations were performed. Autoimmune panel [Anti–Sjögren syndrome A antibody (SS-A); Anti–Sjögren syndrome B antibody (SS-B); antinuclear antibody (ANA); anti-neutrophil cytoplasmic antibody (ANCA); anti-topoisomerase I antibody (Scl-70); anti-histidil-tRNA synthetase antibody (Jo-1); anti-Smith/Ribonucleoprotein antibody (Sm/RNP); anti-Centromer-B antibody; anti-myeloperoxidase antibody (MPO); anti-proteinase-3 antibody (PR3)] showed negative result, and the histopathology findings of the nasal cartilage and mucosa were normal. In the next 8 years, no symptoms occurred other than dyspnea with slow progression. Finally, a computed tomography scan revealed an approximately 10 mm long, subglottic, circumferential soft-tissue mass with a lumen diameter of 4 mm (Fig.1a). No clinical or radiological evidence of the disease was observed outside the larynx. Direct laryngotracheoscopy under general anesthesia with jet ventilation revealed a circumferential fibrotic subglottic stenosis (SGS) (Fig.1b). No other airway abnormality was identified by the endoscopic examination. The patient underwent a slide laryngotracheoplasty as described in an earlier publication (7). Partial midline anterior laryngofissure was performed after the exploration of the laryngotracheal complex and careful dissection of the cricotracheal junction. The cricoid and thyroid cartilage was divided in the midline until the level of the anterior commissure (Fig's 2a and 2b). Then, the plate of the cricoid cartilage was incised in the midline. Increased attention was paid to the preservation of the posterior perichondrium, posterior cricoarytenoid, and pharyngeal constrictor muscle integrity. Laryngeal release and mobilization of the distal trachea were also performed. Accordingly, the anterior cartilage rings of the trachea could be easily pulled up to the level of the anterior commissure. The posterior membranous part of the trachea was sufficiently cropped in order to perfectly fit the posterior subglottic mucosa (Fig.2b). 

Finally, an anastomosis was created between the trachea, anterior cricoid, and midline incised thyroid cartilage with double-armed continuous locked sutures (Fig's 2c, 2d, 2e, and 2f).

**Fig 3:**
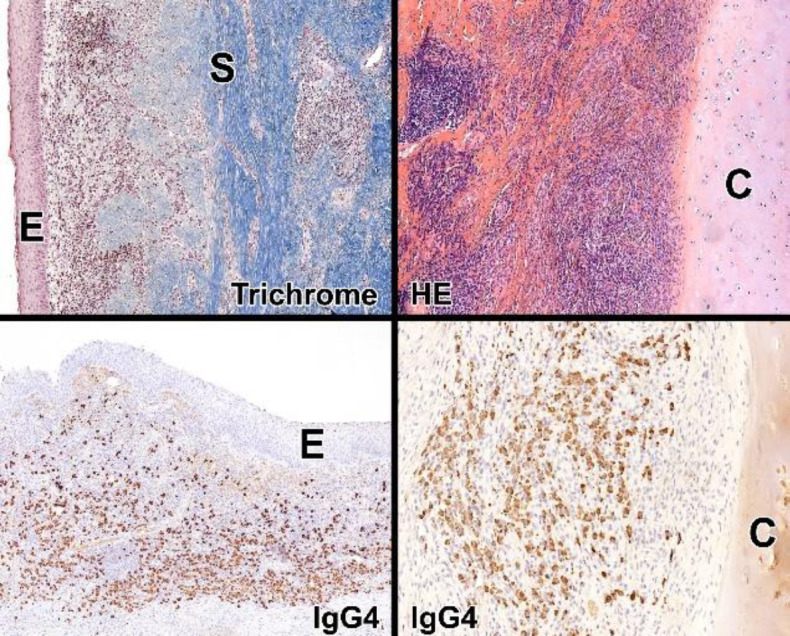
Trichrome-stained tissue section at low-power micrograph: the submucosa (S) thickened by small round cell infiltrates and collagen deposition (blue); the respiratory epithelium replaced by metaplastic squamous epithelium (E)

Histologic findings revealed the replacement of the respiratory epithelium by metaplastic non-dysplastic squamous epithelium. The submucosa was thickened by dense lymphoplasmacytic infiltrates and focally storiform fibrosis. The plasma cell infiltrates proved to be polyclonal, with the abundance of IgG4-positive plasma cells. The ratio of IgG4-positive plasma cells per IgG-positive plasma cell was 75%. Obliterative phlebitis, tissue eosinophilia, or granulomas were not detected (Fig.3).

Hematoxylin-eosin (HE)-stained tissue section at low-power micrograph: storiform pattern of fibrosis and dense infiltrates rich in plasma cells in the deep submucosa; (C): tracheal cartilage

Immunoglobulin G4-related disease (IgG4)-stained tissue sections: at low power magnification, abundant number of IgG4-positive plasma cells under the squamous epithelium (E); a great number of IgG4-positive plasma cells in the deep submucosa; C: tracheal cartilage.

No major perioperative or postoperative complications occurred. The patient was discharged on the 9^th^ postoperative day with good swallowing and breathing. The postoperative ultrasound did not show any fibro-inflammatory disorder in the thyroid and salivary glands. The serum IgG4 level of the patient was 366 mg/l one week after the surgery. In addition, 6 weeks after the intervention, the patient was able to return to her premorbid way of life. During the follow-up period of 22 months, endoscopy revealed no significant restenosis (Figure 1c). The serum IgG4 level did not significantly change (317 mg/l in the 16^th^ postoperative month). 

The peak inspiratory flow increased from 2.3 to 3.8 L/sec (change: 1.54 L/sec; 167% of the baseline) (8). The improvement of the quality-of-life score by 53% (from 15 to 7) also showed the patient’s satisfaction with her respiratory function (9). All the objective voice parameters were within the physiological range in the 22^nd^ postoperative month: Fundamental frequency: 250.1 Hz; Jitter%: 0.33; Shimmer%: 2.82; Harmonics-to-Noise Ratio: 26.2 dB; Maximum Phonation Time: 19.6 s (10). The Voice Handicap Index demonstrated that the patient’s voice also improved (decreased from 20 to 2; 10% of baseline) (11). 

## Discussion

Approximately, 5% of all acquired SGS considered idiopathic after all possible causes (i.e., trauma from intubation, surgery, radiation, autoimmune disorders, infectious processes, and congenital abnormalities) have been ruled out (12). The true etiologies of ISGS have been disputed for decades; nevertheless, it still remains unclear. The pathogenic process is most likely multifactorial. Estrogen and GERD have been theorized to play a role in the development of ISGS (13-15). In addition, a subgroup of ISGS patients may suffer from limited granulomatosis with polyangiitis (Wegener’s granulomatosis). However, the biopsies of airway lesions lack the typical histologic findings and diagnostic serologic markers (16,17). Based on the presented case, IgG4-RD should be kept in mind in case of ISGS. The IgG4-RD can involve virtually any organ or tissue. Therefore, the clinical presentation is very heterogeneous. Some patients present with a single site involved and others may have a few or many organs affected by IgG4-RD. These may develop simultaneously or sequentially. On the other hand, several other diseases (e.g., infections, autoimmune diseases, and cancers) can be associated with the increased levels of IgG4 (4,18,19). Histopathology is a hallmark in the diagnosis of IgG4-RD, with diffuse lymphoplasmacytic infiltrate with an abundance of IgG4-positive plasma cells, storiform fibrosis, with or without obliterative phlebitis and eosinophilia (3,20). An IgG4/IgG tissue plasma cell ratio higher than 40% strongly suggests the disease. Meanwhile, the presence of neutrophils, neutrophilic microabscesses, granulomas, and necrotizing vasculitis does not support the diagnosis of IgG4-RD (21). Elevated serum IgG4 concentration is not necessary for the diagnosis (22). Approximately, 30% of patients with biopsy-proven IgG4-RD have normal serum IgG4 concentrations (1,23,24). 

The IgG4 antibody has a low antigen-binding affinity and accounts for less than 5% of total IgG concentration, with IgG4 concentrations within the range of 0.01-1.4 mg/dL and variability of 5-50x among healthy individuals (24). Important details remain unknown about IgG4-RD, including the pathophysiologic mechanism, exact role of IgG4, and individual organ manifestation. However, head and neck manifestations are quite common, laryngeal involvement was described only in a limited number of cases (3). 

Völker et al. described the first laryngeal manifestation of IgG4-RD in 2009. Since then, isolated subglottic involvement and tracheal and hypopharyngo-laryngeal involvements have been reported (23, 25-30) (Table.1). 

**Table 1:** Published cases of immunoglobulin G4-related disease with laryngotracheal involvement

**Authors**	**Publication**	**Patient**	**Localization**	**Treatment**
	**year**	**gender/age (year)**		
Völker et al.	2009	Male/56	Left false cord	Laser resection and corticosteroid
Virk et al.	2012	Female/22	Subglottic stenosis	Laser resection, dilatation, prednisolone, and
				laryngotracheal reconstruction with costal cartilage graft
Khoo et al.	2013	Male/62	Supraglottic	Prednisolone
Shaib et al.	2013	Male/56	Right anterior subglottic area	Laser resection and tracheotomy
		Male/57	Mucosal hyperplasia of pharynx and larynx	Prednisolone
Kobraei et al.	2013	Female/26	Distal trachea mass	Excision, laser debridement, and tracheal resection
Reder et al.	2014	Male/58	Base of tongue, left aryepiglottic fold,	Laser resection, prednisolone, methylprednisolone, and rituximab
			right vocal process, and left piriform sinus	
		Male/62	Base of tongue, epiglottis, aryepiglottic folds,	Prednisolone, methylprednisolone, and rituximab
			and false and true vocal folds	
Hamadini	2017	Female/54	Postcricoid ulcer	not reported

It is assumed that due to the local IgG4-RD presentation, even short-term intubation was enough for the initiation of the mucosal inflammation process and fibrotic remodeling of the airway mucosa. This might be the way how the former asymptomatic low-grade stenosis became manifest. The typical tumefactive appearance of the lesion remained hidden by the metaplastic non-dysplastic squamous epithelium, caused by the mechanical injury by the endotracheal tube (31). The early diagnosis and effective management of IgG4-RD are crucial for the prevention of sclerotic changes, irreversible organ disfunction, unnecessary medication therapy, and surgical procedures. The treatment heterogeneity in the few reported cases of IgG4-RD with airway involvement is conspicuous. There is no evidence-based treatment protocol from randomized controlled trials. According to the clinical experience, most IgG4-RD patients favorably respond to glucocorticoid (GC) treatment (4,27,28). Reder et al. described the effectivity of anti-CD20 monoclonal antibody rituximab in patients with hypopharyngo-laryngeal involvement (23).

The use of other more intense drugs, such as bortezomib, fludarabine, and cyclophosphamide, which are efficient in patients with other organ involvement, has not yet been investigated in airway patients (31-34). In previously published cases with glottic-subglottic and/or tracheal involvement, GC as a monotherapy (prednisolone and/or methylprednisolone) was not effective (25,27, 28,30,31). Long-standing fibrotic lesions of IgG4-RD generally do not respond well to conservative treatment and surgical resection may be preferred (24). 

In addition, the surgical solution should be primarily chosen in cases of lesions involving critical airway stenosis (29). In case of slide laryngotracheoplasty, a vascularized local trachea flap is used for the augmentation of the subglottic airway. The technique provides an appropriate subglottic airway without graft absorption and donor site reaction. The entire cricoid ring, recurrent nerves, and cricothyroid muscles are preserved, which contribute to the good functional outcomes.

Relapse may develop in the originally affected organs; however, it can also occur in completely different sites. Furthermore, there is no consensus on the systematic follow-up of IgG4-RD patients, and it is still not clear whether IgG4 level can be used for the assessment of treatment response (35). Despite the partial removal of the stenotic lesion and lack of GC therapy, no recurrence was observed during the follow-up in the presented case. Spontaneous remission of IgG4-RD is not unknown; nevertheless, further investigation is required to clarify the triggering and reducing mechanisms of this chronic inflammatory disease (36-38).

## Conclusion

The laryngeal involvement of IgG4-RD is uncommon; nonetheless, it is a manifestation that should be included in the differential diagnosis of SGS. Furthermore, subglottic IgG4-RD might be a potential etiological factor of ISGS and acquired airway stenosis after short-term intubation. Herein, we presented an easily performed reconstruction option using readily available vascularized local tissues. Slide laryngotracheoplasty might be a favorable solution without stenting and tracheostomy even in special cases of SGS.

## References

[1] Kamisawa T, Zen Y, Pillai S, Stone JH (2015). IgG4-related disease. Lancet.

[2] Khosroshahi A, Stone JH (2011). A clinical overview of IgG4-related systemic disease. Curr Opin Rheumatol.

[3] Takano K, Yamamoto M, Takahashi H, Himi T (2017). Recent advances in knowledge regarding the head and neck manifestations of IgG4-related disease. Auris Nasus Larynx.

[4] Pieringer H, Parzer I, Wöhrer A, Reis P, Oppl B, Zwerina J (2014). IgG4- related disease: an orphan disease with many faces. Orphanet J Rare Dis.

[5] Mikulicz J ( 1892). Über eine eigenartige symmetrishe Erkrankung der Tränen und Mundspeicheldrüsen. Billroth T (ed) Beiträge zur Chirurgie.

[6] Yamashita H, Takahashi Y, Ishiura H, Kano T, Kaneko H, Mimori A (2012). Hypertrophic pachymeningitis and tracheobronchial stenosis in IgG4-related disease: case presentation and literature review. Intern Med.

[7] Rovó L, Erdélyi E Zoltán T, Gál P, Szegesdi I, Sztanó B, Sandu K, Bach Á (2020). Slide Laryngotracheoplasty for Congenital Subglottic Stenosis in Newborns and Infants. Laryngoscope.

[8] Cantarella G, Fasano V, Bucchioni E, Domenichini E, Cesana BM (2003). Spirometric and plethysmographic assessment of upper airway obstruction in laryngeal hemiplegia. Ann Otol Rhinol Laryngol.

[9] Jaquet Y, Lang F, Pilloud R, Savary M, Monnier P (2005). Partial cricotracheal resection for pediatric subglottic stenosis: long-term outcome in 57 patients. Thorac Cardiovasc Surg.

[10] Dejonckere PH, Bradley P, Clemente P (2001). A basic protocol for functional assessment of voice pathology, especially for investigating the efficacy of (phonosurgical) treatments and evaluating new assessment techniques. Guideline elaborated by the Committee on Phoniatrics of the European Laryngological Society (ELS). Eur Arch Otorhinolaryngol.

[11] Jacobcon BH, Johnson A, Grywalski C (1997). The voice handicap index (VHI) development and validation. Am J Speech Lang Pathol.

[12] Park SS, Streitz JM, Jr, Rebeiz EE, Shapshay SM (1995). Idiopathic subglottic stenosis. Arch Otolaryngol Head Neck Surg.

[13] Gelbard A, Donovan DT, Ongkasuwan J, Nouraei SA, Sandhu G, Benninger MS (2016). Disease homogeneity and treatment heterogeneity in idiopathic subglottic stenosis. Laryngoscope.

[14] Valdez TA, Shapshay SM (2002). Idiopathic subglottic stenosis revisited. Ann Otol Rhinol Laryngol.

[15] Blumin JH, Johnston N (2011). Evidence of extraesophageal reflux in idiopathic subglottic stenosis. Laryngoscope.

[16] Gluth MB, Shinners PA, Kasperbauer JL (2003). Subglottic stenosis associated with Wegener’s granulomatosis. Laryngoscope.

[17] Stone JH (2003). Limited versus severe Wegener’s granulomatosis: baseline data on patients in the Wegener’s granulomatosis etanercept trial. Arthritis Rheum.

[18] Ebbo M, Grados A, Bernit E, Vély F, Boucraut J, Harlé JR (2012). Pathologies Associated with Serum IgG4 Elevation. Int J Rheumatol.

[19] Sabato V, Vanderveken OM, Van den Wyngaert T, Van Laer C, Ebo D (2017). A patient with a severe glottic stenosis and saddle nose. Acta Clinica Belgica.

[20] Deshpande V, Zen Y, Chan JK, Yi EE, Sato Y, Yoshino T (2012). Consensus statement on the pathology of IgG4-related disease. Mod Pathol.

[21] Deshpande V (2012). The pathology of IgG4-related disease: critical issues and challenges. Semin Diagn Pathol.

[22] Carruthers MN, Khosroshahi A, Augustin T, Deshpande V, Stone JH (2015). The diagnostic utility of serum IgG4 concentrations in IgG4-related disease. Ann Rheum Dis.

[23] Reder L, Della-Torre E, Stone JH, Mori M, Song P (2015). Clinical Manifestations of IgG4-Related Disease in the Pharynx: Case Series and Review of the Literature. Ann Otol Rhinol Laryngol.

[24] Stone JH, Yoh Zen MPH, Deshpande V (2012). IgG4-related disease. N Engl J Med.

[25] Völker HU, Scheich M, Zettl A, Hagen R, Müller-Hermelink HK, Gattenlöhner S (2010). Laryngeal inflammatory myofibroblastic tumors: Different clinical appearance and histomorphologic presentation of one entity. Head Neck.

[26] Virk JS, Stamatoglou C, Kwame I, Salama A, Sandison A, Sandhu G (2012). IgG4-sclerosing pseudotumor of the trachea: a case report and review of the literature. Arch Otolaryngol Head Neck Surg.

[27] Khoo JF, Batt M, Stimpson P, Safdar A (2014). Supraglottic immunoglobulin-G4 related plasma cell granuloma: case report and literature review. Head Neck.

[28] Shaib Y, Ton E, Goldschmeding R, Tekstra J (2013). IgG4-related disease with atypical laryngeal presentation and Behçet/granulomatous polyangiitis mimicking features. BMJ Case Rep.

[29] Kobraei EM, Song TH, Mathisen DJ, Deshpande V, Mark EJ (2013). Immunoglobulin g4-related disease presenting as an obstructing tracheal mass: consideration of surgical indications. Ann Thorac Surg.

[30] Hamadani S, Wang B, Gupta S (2018). IgG4-related disease presenting as hoarseness and postcricoid ulcer. Ann Allergy Asthma Immunol.

[31] Hom DB, Hebda P, Friedman C, Gosai A (2006). Essential Tissue Healing of the Face and Neck.

[32] Bosco JJ, Suan D, Varikatt W, Lin MW (2013). Extra-pancreatic manifestations of IgG4-related systemic disease: a single-centre experience of treatment with combined immunosuppression. Intern Med J.

[33] Wong PC, Fung AT, Gerrie AS, Moloney G, Maberley D, Rossman D (2013). IgG4-related disease with hypergammaglobulinemic hyperviscosity and retinopathy. Eur J Haematol.

[34] Khan ML, Colby TV, Viggiano RW, Fonseca R (2010). Treatment with bortezomib of a patient having hyper IgG4 disease. Clin Lymphoma Myeloma Leuk.

[35] Tabata T, Kamisawa T, Takuma K, Egawa N, Setoguchi K, Tsuruta K (2011). Serial changes of elevated serum IgG4 levels in IgG4-related systemic disease. Intern Med.

[36] Yamakawa H, Sekine A, Yamanaka Y, Sadoyama S, Baba T, Hagiwara E (2017). Pathologically Proven Spontaneous Remission of IgG4-related Retroperitoneal Fibrosis. Intern Med.

[37] Kase S, Yamamoto T, Ishijima K, Noda M, Ishida S (2013). Spontaneous regression of IgG4-related dacryoadenitis. Mod Rheumatol.

[38] Raj R (2013). IgG4-related Lung Disease. Am J Respir Crit Care Med.

